# The complete chloroplast genome of *Wurfbainia neoaurantiaca* (Zingiberaceae: Zingiberea) from Yunnan Province, China

**DOI:** 10.1080/23802359.2021.1938722

**Published:** 2021-06-30

**Authors:** Chunyong Yang, Yanqian Wang, Yanfang Wang, Shuang Li, Ge Li, Zhen Yan, Lixia Zhang, Jianjun Qi

**Affiliations:** a Yunnan Key Laboratory of Southern Medicinal Utilization, Yunnan Branch Institute of Medicinal Plant Development, Chinese Academy of Medical Sciences and Peking Union Medical College, Jinghong, China; b Institute of Medicinal Plant Development, Chinese Academy of Medical Sciences and Peking Union Medical College, Beijing, China

**Keywords:** Complete chloroplast genome, phylogenetic analysis, *Wurfbainia neoaurantiaca*

## Abstract

*Wurfbainia neoaurantiaca* is a medicinal plant endemic to Yunnan Province, China. In this study, its complete chloroplast genome was assembled and characterized. The total genome size of *W. neoaurantiaca* was 158,484 bp in length, consisting of a large single-copy region (LSC), a small single-copy region (SSC) and two inverted repeat regions (IRs) with 88,605 bp, 15,285 bp and 29,822 bp, respectively. Its GC content was 36.08%. The chloroplast genome encoded 113 unique genes, including 79 protein-coding, 30 tRNA, and four rRNA genes. The result of the phylogenetic analysis indicated that *W. neoaurantiaca* was related to *W. villosa* var. *xanthioides* and supported de Boer’s classification that *W. compacta*, *W. longiligularis*, *W. neoaurantiaca*, *W. villosa*, *W. villosa* var. *xanthioides* and *Amomum krervanh* belonged to the *Wurfbainia* Clade.

*Wurfbainia neoaurantiaca* (former Latin names: *Amomum aurantiacum* H. T. Tsai & S. W. Zhao, 1979, *Amomum neoaurantiacum* T. L. Wu, K. Larsen & N. J. Turland, 2000) is a perennial medicinal herb classified in the family Zingiberaceae, which is endemic to the Yunnan Province of China (de Boer et al. 2018; Wu and Turland 2001). Its fruits are used as herbal medicine and have been used to treat gastrointestinal diseases (Ma et al. 2018). It is difficult to distinguish *W. neoaurantiaca* from its related species such as *Wurfbainia. villosa*, *W. villosa* var. *xanthioides* and *W. longiligularis* due to their similar morphological characters. In recent years, the chloroplast genome as a super-barcode has been shown to be an efficient method to distinguish species (Abdullah et al. 2019; Cui et al. 2019; Liang et al. 2019), and phylogenetic analysis of chloroplast genomes has been successfully used to identify *Wurfbainia* species (Cui et al. 2019). In this study, the chloroplast genome of *W. neoaurantiaca* was assembled, annotated, characterized, and a phylogenetic analysis was performed to investigate its evolutionary relationship with other *Wurfbainia* species and confirm its phylogenetic position in the family location.

The plants of *W. neoaurantiaca* were collected from Jinghong (100°52′44ʺE; 21°48′56ʺN), Yunnan Province, China. Voucher specimen (Y20180726015) and its DNA (DNA-Y20180726015) were deposited in the Herbaria and Medicinal Plant Cultivation Research Center of Yunnan Branch Institute of Medicinal Plant Development, Chinese Academy of Medical Sciences and Peking Union Medical College, respectively (www.yn-implad.ac.cn, contacts: Dr. Haitao Li, email contact: lhtxyl@126.com). The genomic DNA was extracted from the leaves using the DNeasy Plant Mini Kit (Qiagen, Dusseldorf, Germany). The quality of the DNA was assessed using a Nanodrop One^C^ spectrophotometer (Thermo Scientific, Madison, USA) and determined by 1.5% agarose gel electrophoresis.

A paired-end library of 350 bp was constructed and sequenced on the NovaSeq system (Illumina, San Diego, USA). A total of 8.5 Gb reads were generated and used to assemble the chloroplast genome using the default settings in NOVOplasty 4.0 (Dierckxsens et al. 2017). Annotation of the chloroplast genome was executed using GeSeq (Tillich et al. 2017) and corrected manually.

The chloroplast genome sequence of *W. neoaurantiaca* is 163,534 bp in length and exhibits a general quadripartite structure containing a large single-copy region (LSC), a small single-copy region (SSC) and two inverted repeat regions (IRs) with 88,605 bp, 15,285 bp and 29,822 bp, respectively. The GC content is 36.08%. The chloroplast genome of *W. neoaurantiaca* encodes 113 unique genes, including 79 protein-coding, 30 tRNA and four rRNA genes, in which 10 genes (*atpF*, *ndhA*, *ndhB*, *petB*, *petD*, *rpoC1*, *rps12*, *rps16*, *rpl2*, *rpl16*) contain one intron, and two genes (*clpP* and *ycf3*) contain two introns. Relative synonymous codon usage (RSCU) was calculated with CodonW 1.4.4 (https://sourceforge.net/projects/codonw/files/) to reveal synonymous codon bias in the coding sequences. Except for *trnL*-CAA encoded by UUG and termination codon UAA, 28 amino acid codons with RSCU value > 1 ended with A/U. Simple sequence repeats (SSRs) were mined using MISA-web (https://webblast.ipk-gatersleben.de/misa/), and up to 153 SSRs were detected in the whole chloroplast genome, including 94 mono-, 32 di-, five tri-, 16 tetra-, four penta-, and two hexa-nucleotide SSRs.

The chloroplast genome sequences of 23 species (including *W. neoaurantiaca*) from Zingiberaceae and three outgroup taxa from the Musaceae were aligned with MAFFT 7.307 (Katoh and Standley 2013). A phylogenetic tree was constructed using RAxML 8.2.12 (Stamatakis 2014) with 1000 Bootstrap replicates and using the GTR + F+R2 model according to ModelFinder (Kalyaanamoorthy et al. 2017). The result indicated that the selected species from the Zingiberaceae were clustered within a lineage distinct from the outgroup. Five *Wurfbainia* species and *Amomum krervanh* formed a monophyletic clade in the Zingiberaceae. *Wurfbainia neoaurantiaca* was fully resolved in a clade with *W. longiligularis*, *W. villosa* var. *xanthioides*, *W. villosa* and more closely related to *W. villosa* var. *xanthioides* (Figure 1). According to the recircumscription of the genus *Amomum*, *W. compacta*, *W. longiligularis*, *W. neoaurantiaca*, *W. villosa*, *W. villosa* var. *xanthioides* and *Amomum krervanh* were classified into the *Wurfbainia* Clade (de Boer et al. 2018). Our results confirm the classification and further illustrate the relationship among *W. neoaurantiaca* and the other five species.

**Figure 1.**
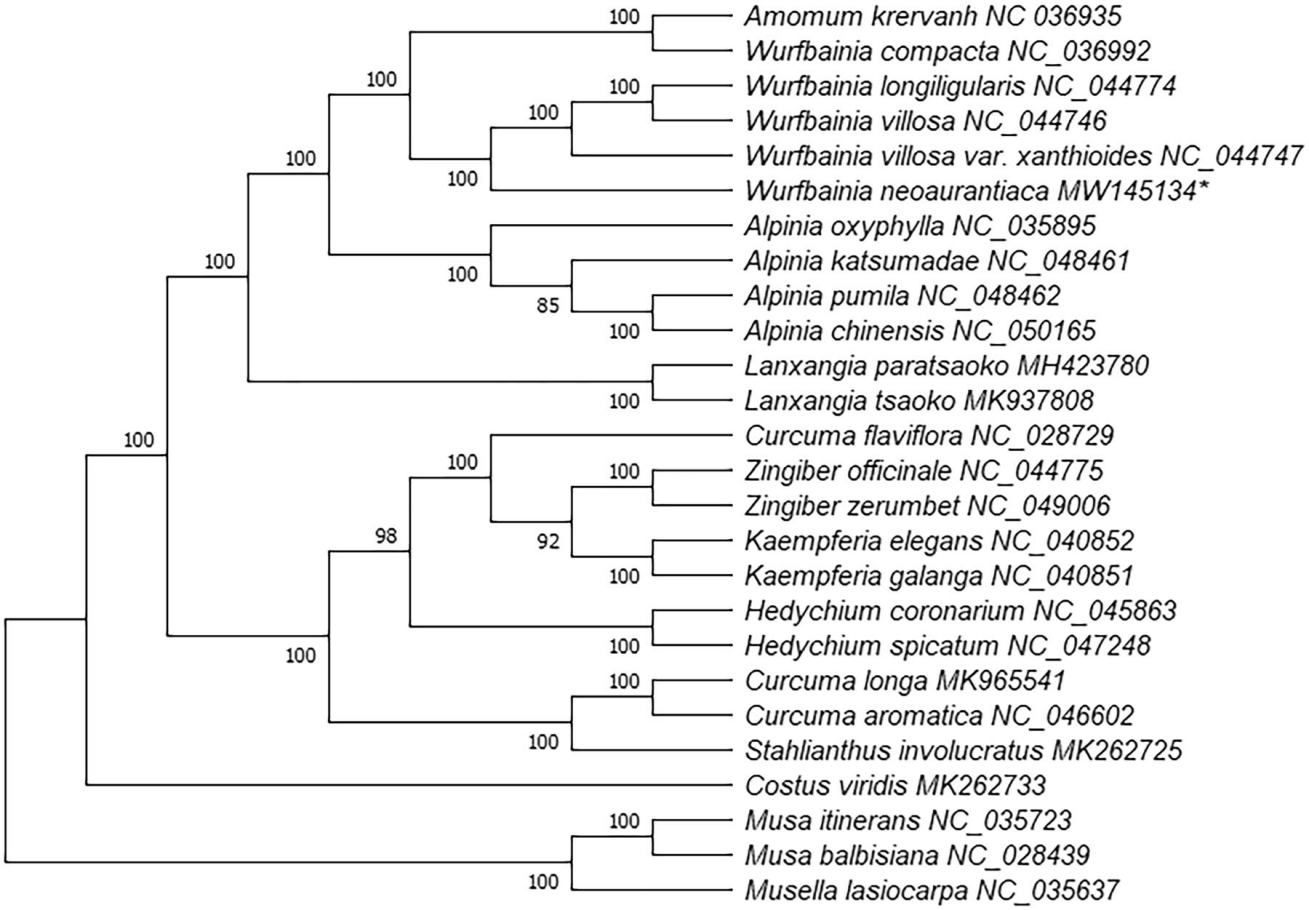
A phylogenetic ML tree based on the complete chloroplast sequences of 23 Zingiberaceae species and three Musaceae species.

## Data Availability

The genome sequence data that support the findings of this study are openly available in GenBank of NCBI at https://www.ncbi.nlm.nih.gov/ under the accession no. MW145134. The associated BioProject, Bio-Sample and SRA numbers are PRJNA699991, SAMN17817191 and SRP304958, respectively.
